# Plasticized PVC‐Gel Single Layer‐Based Stretchable Triboelectric Nanogenerator for Harvesting Mechanical Energy and Tactile Sensing

**DOI:** 10.1002/advs.202201070

**Published:** 2022-05-26

**Authors:** Hyosik Park, Seung‐Ju Oh, Daeyeong Kim, Mingyu Kim, Cheoljae Lee, Hyeonseo Joo, Insun Woo, Jin Woo Bae, Ju‐Hyuck Lee

**Affiliations:** ^1^ Department of Energy Science and Engineering Daegu Gyeongbuk Institute of Science and Technology (DGIST) Daegu 42988 Republic of Korea; ^2^ Future Convergence Engineering School of Energy Materials and Chemical Engineering Korea University of Technology and Education 1600, Chungjeol‐ro Cheonan 31253 Republic of Korea; ^3^ Energy Science and Engineering Research Center Daegu Gyeongbuk Institute of Science and Technology (DGIST) 333 Techno Jungang‐daero, Hyeonpung‐eup, Dalseong‐gun Daegu 42988 Republic of Korea

**Keywords:** energy harvesting, polyvinyl chloride gel, tactile sensor, triboelectric nanogenerator

## Abstract

Triboelectric nanogenerators have garnered significant attention as alternative power sources for wearable electronics owing to their simple structure, easy fabrication, low cost, and superior power output. In this study, a transparent, stretchable, and attachable triboelectric nanogenerator (TENG) is built with an advanced power output using plasticized polyvinyl chloride (PVC)‐gel. The PVC‐gel exhibit very high negative triboelectric properties and electrically insulating PVC became an electrically active material. It is found that a single layer of PVC‐gel can act as a dielectric and as a conducting layer. The PVC‐gel based single layer of triboelectric nanogenerator (S‐TENG) creates output signals of 24.7 V and 0.83 µA, i.e., a 20‐fold enhancement in the output power compared to pristine PVC‐based TENGs. In addition, the S‐TENG can stably generate output voltage and current under stretching condition (80%). The S‐TENG can be implemented as a tactile sensor that can sense position and pressure without combining multiple elements or electrode grid patterns. This study provides new applications of power sources and tactile sensors in wearable electronics.

## Graphical abstract

A plasticized polyvinyl chloride (PVC)‐gel has enhanced mechanical and electrical properties than native PVC in triboelectric effect. The stretchable single layer triboelectric nanogenerator (S‐TENG) can work because of the enhanced stretchability and conductivity of the plasticized PVC‐gel. The mechanism of generating output in the single layer TENG can help it sense the contact position and pressure as a tactile sensor.

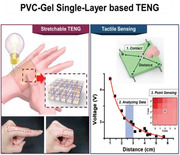

## 1 Introduction

In addition to rigid or flexible frame‐based electronics, the development of stretchable electronics for use in wearable/attachable electronics or electronic/ionic skin,^[^ ^1^, ^2^, ^3^, ^4^ ^]^ comprising transistors,^[^ ^5^, ^6^ ^]^ luminescence devices,^[^ ^7^, ^8^ ^]^ smart sensors,^[^ ^9^, ^10^, ^11^ ^]^ actuators,^[^ ^12^, ^13^ ^]^ bioapplications,^[^ ^14^, ^15^, ^16^ ^]^ and energy devices^[^ ^17^, ^18^ ^]^ has received significant attention. These devices need to be thin, light, stretchable, attachable, and transparent to be integrated with clothes or attached to or implanted into a curved human body. However, it is challenging to meet these requirements while maintaining the power supply because current batteries or capacitors, which are the most commercialized portable power sources, are relatively heavy and face critical lifetime limitations if they are made smaller or flexible. A sustainable and alternative power source for portable electronics, triboelectric nanogenerators (TENGs), which convert mechanical energy into electrical energy, have received considerable attention because of their simple structure, multiple material options, low cost, easy fabrication, and superior power output performance.^[^ ^19^, ^20^ ^]^ Recently, the development of stretchable and transparent TENGs using silicone elastomer as a stretchable dielectric material^[^ ^21^, ^22^, ^23^ ^]^ and hydrogel, ion‐gel, and physiological saline as a stretchable electrode^[^ ^24^, ^25^, ^26^, ^27^, ^28^ ^]^ has been reported. These electrodes could help solve the challenge of serving both high stretchability and transmittance in TENGs. However, the method of robust encapsulation via the chemical anchoring process, which is performed to protect the stretchable electrode from leakage, dehydration, and contamination, compromises the mechanical stability and integration of the TENGs with other electronic devices.^[^ ^29^, ^30^ ^]^

The structure of a typical TENG is composed of two or more layers of substrate/electrode/dielectric, substrate/electrode, or electrode/dielectric, resulting in a relatively complex fabrication process and low mechanical robustness. Herein, we report a plasticized polyvinyl chloride (PVC)‐gel based single‐layered, stretchable, attachable, and transparent TENG that can harvest biomechanical energy and can be used for tactile sensing.^[^ ^31^ ^]^ The TENG was fabricated using PVC and an adipate‐based plasticizer. The plasticizer (500 µm thickness) improved the transparency (up to 91% at a wavelength of 550 nm) and stretchability (250%) of the intrinsically opaque and rigid PVC. As the amount of plasticizer in the PVC‐gel increased, the PVC‐gel exhibited not only a highly improved dielectric constant (90–300 times higher than intrinsic PVC) but also a highly negative triboelectric behavior (more negative than perfluoroalkoxy, PFA) that contributed to the enhanced TENG performance. Furthermore, the added plasticizer improves the electrical conductivity, making PVC‐gel acts not only as a dielectric but also as an electrode, thereby enabling the development of single layer TENGs (S‐TENGs). The developed PVC‐gel based S‐TENG generates electricity by simply contacting various materials, including nylon, polyethylene terephthalate (PET), polyethylene naphthalate (PEN), and a human finger. Unlike conventional tactile sensor,^[^ ^32^ ^]^ PVC‐gel based tactile sensor can sense position and pressure without electrode grid patterning, thereby taking advantage of the high flexibility/stretchability and transparency of the PVC‐gel. The current study presents a single layer PVC‐gel based TENG for the first time, allowing energy generation and tactile sensing without complex and expensive electrode layer/patterning. Therefore, this TENG can be potentially used in electronic skins, soft robots, smart displays, wearable electronics, etc.

## 2 Results and Discussions

**Figure 1:**
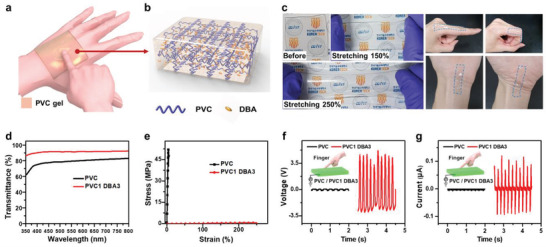
Properties of the PVC‐gel. a) Schematic of the PVC‐gel based single layer TENG. b) 3D network structure of the PVC‐gel. c) Photograph of the stretchable (150%, 250%) and conformal contact to the bent finger and wrist of PVC‐gel. d) Transmittance and e) stress–strain curves of PVC and PVC‐gel. f,g) Output voltage and current of the S‐TENG by touching PVC and PVC‐gel with a finger.

**Figure** **1**a shows a schematic of the PVC‐gel based S‐TENG. The PVC‐gel was prepared by simply mixing PVC and the plasticizer dibutyl adipate (DBA) (Figure 1b). The experimental procedures are described in detail in the Experimental Section. The Fourier transform infrared spectroscopy (FT‐IR) spectra for the only PVC, DBA, and PVC‐gel confirmed that the DBA plasticizer was physically distributed in the PVC chain without forming specific intermolecular bonds (Figure S1 and Note S1, Supporting Information). Figure 1c shows images of a PVC‐gel that exhibits high transparency to all visible colors, high stretchability of over 250%, and high conformal adhesion to delicate curved skin. Besides, the PVC‐gel does not show any degradation and weight loss at room temperature without encapsulation for 30 days (Figure S2, Supporting Information). The 500 µm thick PVC‐gel achieves a transmittance of 91% in the visible range (wavelength 550 nm), which is higher than that of the intrinsic PVC (78% at 550 nm) (Figure 1d). Uniaxial tensile tests were performed to evaluate the mechanical properties of the original PVC and the plasticizer‐treated PVC‐gel (Figure 1e). The native PVC exhibited a linear elastic behavior with ultimate stress of 50 MPa, Young's modulus of 1.3 GPa, and elongation at a break of 4%. By contrast, the PVC‐gel (PVC1 DBA3) displayed a ductile behavior with the stress of 1 MPa, Young's modulus of 287 kPa, and elongation at a break of 250% (Figure S3, Supporting Information). The results depict that the plasticized PVC‐gel exhibited high stretchability and transmittance, which are attributed to the physically cross‐linked 3D PVC polymer network in the PVC‐gel. The end chains of the colorless liquid‐state DBA plasticizer widened the gap between the pure PVC chains and thus introduced an additional free volume into the PVC chains. This resulted in a disordered arrangement and sufficient entanglement of the PVC chains, creating many microcrystallites in the amorphous region of the PVC‐gels with a physically cross‐linked 3D network structure. These microcrystallites, which is an average interlayer spacing of 44 nm, were too thin and insufficient to scatter visible light (390–780 nm), resulting in the formation of highly transparent PVC‐gels.^[^ ^12^ ^]^ Additionally, the entangled polymer chains, which acted as microcrystallites in the network, enhanced the ductility of the PVC‐gels. Figure 1f,g shows the output voltage and current of the only PVC and PVC‐gel generated by the finger‐touch‐induced triboelectric effect. The output voltage and current of PVC and PVC‐gel were measured to be 0.3 V, 0.016 µA, and 6 V, 0.2 µA, respectively. The PVC‐gel based TENG exhibits an enhancement in the output performance, i.e., ≈20 times improvement in voltage and 12 times improvement in current.

**Figure 2:**
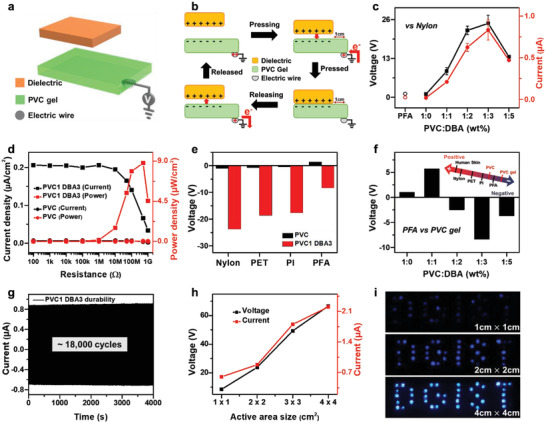
Properties of the S‐TENG. a) Schematic of device structure of S‐TENG. b) The working mechanism of S‐TENG. c) Output voltage and current of S‐TENG as a function of DBA contents. d) Output current density and power density of the PVC and PVC1 DBA3‐based S‐TENG as a function of external load resistance. e) Comparison of triboelectricity between PVC, PVC1 DBA3, and other polymers (Nylon, PET, PI, and PFA). f) Triboelectricity of PVC‐gels compare to PFA and its position in triboelectric series (inset). g) Durability test of PVC1 DBA3‐based S‐TENG for more than 18 000 cycles. h) Output voltage and current according to the active area size of S‐TENG and i) the photograph of LED emitting light accordingly.

To systematically investigate the PVC‐gel's triboelectricity, we first fabricated a PVC‐gel single layer based single electrode vertical contact mode S‐TENG, as shown in **Figure** **2**a. The S‐TENG consists of a 2.5 cm × 3 cm PVC or PVC‐gel film with an electric wire connected for electrical connection. The output performances of the S‐TENG were measured in an active area of 2 cm × 2 cm. The frequency of the contact–separation motion and the pressure between the two contacting films were maintained at 5 Hz and 50 kPa, respectively, for all subsequent tests. Figure 2b shows the working mechanism of the single layer TENG based on PVC‐gel. The mechanism can be interpreted by the combined effect of contact electrification and electrostatic forces, discussed in Note S2 of the Supporting Information. First, we measured the output performance of the PVC and PVC‐gel based S‐TENG as a function of the DBA/PVC weight ratio ranging from 0 to 5 using a nylon film (Figure 2c; Figure S4, Supporting Information). The result shows that the triboelectric output performance increases until the weight ratio reaches 3, reaching a maximum value of ≈24.7 V and 0.83 µA, and then decreases with a further increase in the DBA content. The generated output voltage and current are 24.7 times higher than that of a native PVC film with an output voltage of 1 V and an output current of 19 nA and are 20.5 times higher than that of a PFA film, the most representative negative triboelectric material with an output voltage of 1.2 V and an output current of 24 nA. The instantaneous power densities were also obtained by measuring the output current of the PVC and PVC‐gel based (PVC1 DBA3) S‐TENG with an external load resistance ranging from 100 Ω to 1 GΩ, as shown in Figure 2d. The maximum output power densities of the PVC film and PVC‐gel S‐TENG were 28 nW cm^−2^and 8.7 μW cm^−2^, respectively, with a 311‐fold enhancement in the output power density at 500 MΩ. The performance measurement of the double‐electrode type TENG confirmed the similarity in its behavior, and it is discussed in the supporting information (Figures S5 and S6, and Note S3, Supporting Information). The DBA plasticizer molecules in PVC‐gel act as a medium of the electric field and allow PVC‐gel to act as a conducting layer.^[^ ^33^ ^]^ As a result of comparing the output voltage according to the presence or absence of the ITO electrode, it was proved that PVC‐gel exhibits relatively high electrical resistance compared to a conventional electrode but still acts as an electrode for TENG (Figure S7, Supporting Information). We also measured the output voltage and current of the S‐TENG concerning the pressure and frequency, and it is discussed in the supporting information (Figures S8 and S9, and Note S4, Supporting Information).

In addition, we measured the output voltage of S‐TENG based on PVC and PVC‐gel (PVC1 DBA3) based on various dielectric materials, such as nylon, PET, PI, and PFA, to identify the position in the triboelectric series (Figure 2e). PVC exhibits more tribonegative properties compared to nylon, PET, and PI as it gains electrons by contact electrification, and it shows negative triboelectric voltage; however, it is more tribopositive compared to PFA. PVC loses electrons by contact electrification with PFA, and the polarization of the *V*_oc_ is reversed, as expected based on the established triboelectric series table.^[^ ^34^ ^]^ By contrast, PVC‐gel (PVC1 DBA3) exhibits more tribonegative properties than all‐dielectric layers used, such as nylon, PET, PI, and PFA. PVC‐gel loses electrons by contact electrification with other dielectric layers, and therefore, they generate all negative voltages. In addition, we compared triboelectric properties of the PVC‐gel as a function of the DBA/PVC ratio with PFA (Figure 2f; Figure S10, Supporting Information). When the PFA film contacts the native PVC and PVC1 DBA1, they generate a positive output. However, when it contacts PVC‐gel with a DBA ratio of over 2, they generate a negative output, implying that the PVC‐gel with a DBA ratio of over 2 exhibits tribonegative properties compared to PFA. The reason that PVC‐gel shows more tribonegative properties as the concentration of DBA increases is that the density of C═O groups with high electronegativity increases.^[^ ^35^ ^]^ The surface potentials of PVC and PVC‐gels measured using the Kelvin probe force microscopy (KPFM) also confirmed a more negative potential for the PVC‐gel than for the native PVC (Figure S11, Supporting Information). According to the empirical results of PVC and PVC‐gel‐based S‐TENGs, we obtained the position of the PVC‐gel in the triboelectric series (in the inset of Figure 2f), which shows that PVC‐gel exhibits extremely negative triboelectric properties, indicating a significant improvement in the output power. Furthermore, we performed a long‐term durability test of the S‐TENG to evaluate its practical applicability. The output current showed no apparent degradation after 3600 s (more than 18 000 cycles) of repeated contact–separation motion of the S‐TENG with a nylon film (Figure 2g). In addition, the S‐TENG can generate strong electrical energy that can function as a power source. Figure 2h shows the measured output voltage and current concerning the active area of the S‐TENG. As the size of the active area increases, the output power also increases. Thus, it can generate sufficient electrical energy to drive 39 blue LEDs by simple contact–separation motion (Figure 2i).

**Figure 3:**
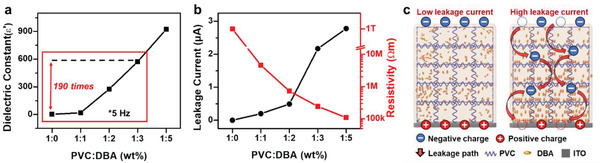
Dielectric constant and leakage current of the PVC‐gel. a) Dielectric constant of PVC‐gel according to PVC:DBA ratio. b) Leakage current and electrical resistivity of PVC‐gel according to PVC:DBA ratio. c) Schematics of the surface charge dissipation induced by the leakage current of the PVC‐gel.

The performance of the TENG is not only affected by the triboelectric series of the two contact materials but also by their electrical properties. The TENG is based on the principle of a parallel‐plate capacitor, which exhibits a relationship between the electric field and the dielectric constant. The triboelectric output is proportional to the surface charge density of the dielectric material. This is expressed in the following equation^[^ ^36^ ^]^

(1) $$V_{\mathrm{OC}}=\frac{\sigma x\left(t\right)}{\varepsilon_{0}}$$

where *σ* is the total surface charge density and is affected by the dielectric constant of the material according to the equation given below^[^ ^37^, ^38^ ^]^

(2) $$\sigma =\frac{\sigma_{0}x\left(t\right)}{x\left(t\right)+d_{\text{PVC}\text{gel}}/\varepsilon_{\text{PVC}\text{gel}}}$$

where *ε*_PVC gel_ and *d*_PVC gel_ are the dielectric constant and thickness of the PVC‐gel films, respectively, *x*(*t*) represents the gap distance between the PVC‐gel and dielectric material, and *σ*_0_ is the triboelectric charge density at the equilibrium state. Thus, the enhanced dielectric constant can increase the total charge density (*σ*) and triboelectric output performance. We measured the dielectric constants of the native PVC and PVC‐gels (**Figure** **3**a) as a function of the plasticizer concentration. The dielectric constant drastically increases with an increase in the plasticizer concentration. The dielectric constants of PVC1 DBA3 and PVC1 DBA5 are 572 and 923, which is 190 and 307 times higher than that of native PVC at 5 Hz, respectively. We also observed that the dielectric constant increases in all frequency ranges (Figure S12, Supporting Information).^[^ ^33^ ^]^ The dipole on the PVC chain could not follow the changes in the electric field polarity because of the structural features of the native PVC. However, the DBA molecules in the PVC‐gels could get charged and polarized under an electric field, facilitating a free dipole reorientation of the PVC chain segments in the PVC‐gel networks in a limited frequency range. Therefore, we concluded that the PVC‐gel could exhibit a high dielectric constant due to polarity's reorientation.^[^ ^39^ ^]^ It is believed that an enormous amount of electrified DBA led to a stronger dipole reorientation of the PVC molecules in the PVC‐gel network, thereby exhibiting a gradual increase in the relative permittivity of PVC‐gel concerning an increase in DBA concentration. Therefore, the PVC‐gel with a large amount of DBA is expected to generate a higher output performance in the TENG. However, as the plasticizer increases, the electrical resistivity of PVC‐gel decreases, and the leakage current increases (Figure 3b; Figure S13, Supporting Information). The surface charge density decreased when the electrical conductivity of the material increased, because of the increase in the charge dissipation rate, as shown below^[^ ^40^, ^41^ ^]^

(3) $$\sigma =\sigma_{T}-\sigma_{D}$$

where *σ*_T_ is the triboelectric effect‐induced surface charge density due to contact electrification between two dielectric materials, and *σ*_D_ is the dissipated surface charge density. The native PVC exhibits a high resistivity (>1 TΩ m) and negligible leakage current. Contrarily, PVC‐gels treated with DBA exhibit low resistivity [4.62 MΩ m (1:1), 725 kΩ m (1:2), 242 kΩ m (1:3), and 109 kΩ m (1:5)] and a relatively high leakage current [0.2 µA (1:1), 0.49 µA (1:2), 2.17 µA (1:3), and 2.78 µA (1:5)]. This suggests that the dipoles induced in the PVC‐gel under the influence of an electric field do not strongly accumulate onto the gel surface but are instead dissipated easily.^[^ ^18^ ^]^ Thus, a large amount of DBA caused an increase in the charge dissipation rate and consequently decreased the output performance of the TENG (Figure 3c). Therefore, the output performance of the PVC‐gel‐based S‐TENG increased with an increase in the DBA content up to a 1:3 ratio owing to the triboelectric property and dielectric constant improvement and decreased subsequently for higher ratios of DBA contents because of the increased charge dissipation rate (Figure 3a,b; Figure S10, Supporting Information)

**Figure 4:**
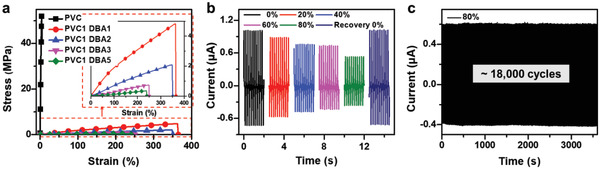
Performance of the S‐TENG in the stretched state. a) Stress–strain curves of PVC‐gel as a function of the PVC/DBA ratio. b) S‐TENG output current from 0% to 80% stretched state and recovered state. c) Durability test of S‐TENG (PVC1 DBA3) at 80% stretched state for more than 18 000 cycles.

Next, we investigated the stretchability of the PVC‐gel based S‐TENG. As shown in the stress–strain curves of the PVC‐gel with a PVC:DBA ratio of 1:0 to 1:5, the Young's modulus decreased (i.e., became softer) as the amount of DBA increased (**Figure** **4**a). The Young's modulus of PVC‐gel obtained with PVC:DBA ratios of 1:0, 1:1, 1:2, 1:3, and 1:5 was 1.3 GPa, 1.6 MPa, 0.66 MPa, 0.28 MPa, and 0.14 MPa, respectively (Figure S3 and Note S5, Supporting Information). This is attributed to the plasticization of the rigid PVC chains, inducing an increase in the distance between the PVC chains and thus, decreasing the interaction force, which further enhances the PVC chain mobility. The elongation at the break of the plasticized PVC‐gels also increased tremendously. In addition, stress–strain hysteresis loops of the cyclic test (10 cycles) for PVC1 DBA3 almost completely overlapped with each other (Figure S14, Supporting Information). No apparent decrease in the stress at the strain of 200% is detected. The viability of the PVC‐gel (1:3) S‐TENG at stretched states in harvesting mechanical energy was further evaluated. The S‐TENG was uniaxially stretched for different stretches or strains, and the corresponding electrical outputs were recorded for a contact–separation motion relative to a nylon film (Figure 4b; Figure S15, Supporting Information). Compared with the initial state (without strain, 0%), the output power of the S‐TENG slightly decreased after being stretched from 20% to 80%. In the stretched state, the entangled PVC‐gel molecules stretched and loosened, resulting in a decrease in the surface charge density as compared to before stretching for the same active area (2 cm × 2 cm).^[^ ^42^ ^]^ In addition, the electrical resistivity of PVC‐gel slightly increased in the stretched state (Figure S16, Supporting Information). When it was released again, the output recovered back to the initial state. The durability of the S‐TENG in the stretched (80%) state was also investigated over long‐term motion cycles. S‐TENGs in the initial and stretched states operated stably, as the short‐circuit current did not show any noticeable degradation after ≈3600 s (more than 18 000 cycles) of repeated contact–separation motion (Figure 4c).

**Figure 5:**
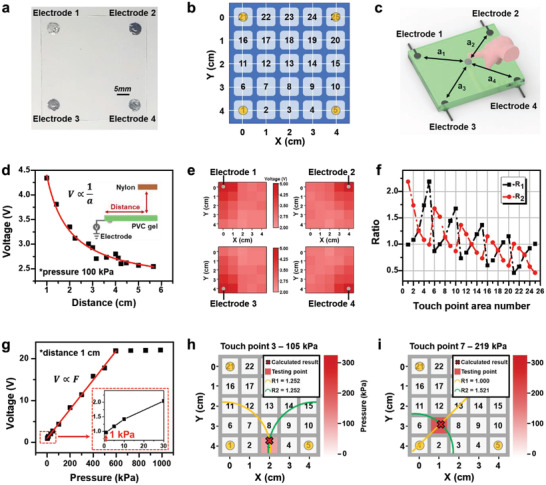
Properties of the S‐TENG based tactile sensor. a) Photograph of PVC‐gel tactile sensor. b,c) Schematics of PVC‐gel tactile sensor with 4‐point electrodes. d) Output voltage of S‐TENG tactile sensor as a function of the distance between contact position and electrode with 100 kPa of pressure. e) Heat map of an output voltage of the S‐TENG tactile sensor according to contact position at each electrode. f) Ratio of generated voltage R1 and R2 at each touch point of S‐TENG tactile sensor. g) Output voltage according to the applied pressure at 1 cm to the electrode. h,i) Position and pressure sensing demonstration of S‐TENG based tactile sensor at the touch point 3 with 105 kPa of pressure and the touch point 7 with 219 kPa of pressure.

The output of the TENG is generally utilized to sense the pressure using the active contact area changing with respect to the applied pressure, and the contact position can be specified by the integration of multiple devices or multiple electrode grid patterns, as reported in several previous reports.^[^ ^43^, ^44^ ^]^ In this study, we propose a new type of tactile sensor using S‐TENG and 4‐point electrodes at the edge of the PVC‐gel that can sense position and pressure without electrode grid patterning, as shown in **Figure** **5**. The PVC‐gel (5 cm × 5 cm) was prepared, and 4‐point circular electrodes with a diameter of 5 mm were deposited on the bottom edge of the PVC‐gel, as shown in the photo image (Figure 5a). The touch position was identified by marking the number on the horizontal and vertical scales, as shown in the schematic in Figure 5b. Figure 5c shows the schematic of distances on a tactile sensor between the touch position and each electrode. First, we found a distance‐sensitive output voltage of the S‐TENG because the electric displacement field depends on the contact position's distance and the relatively high electric resistance of the PVC‐gel.^[^ ^45^ ^]^ As shown in Figure 5d, the output voltage, as a function of the distance between the touch point and electrode, is inversely proportional to the distance. The open‐circuit voltage based on the distances of the S‐TENG can be expressed as

(4) $$V_{\mathrm{OC}}\left(n\right)\propto k\frac{S\sigma }{\sqrt{h^{2}+a_{n}^{2}}},n=1,2,3,4$$

where *h* is the gap distance, *a_n_* is the distance of the contact point on the PVC‐gel to the electrode “*n*”, *S* is the active area, and *k* is the Coulomb force constant, which are expressed in detail in Figure S17 and Note S6 (Supporting Information). Figure 5e shows the output voltage collected from point electrodes 1 to 4 in a heat map form. As the touch position is closer to the electrode, the output voltage is larger, and the output voltage decreases with an increase in the distance.

According to Equation (4), the ratio of the electric potentials of *V*_oc_(1) to *V*_oc_(4) can be expressed as^[^ ^46^ ^]^

(5) $$\frac{V_{\mathrm{OC}}\left(4\right)}{V_{\mathrm{OC}}\left(1\right)}\approx \frac{k\frac{S\sigma }{\sqrt{h^{2}+a_{4}^{2}}}}{k\frac{S\sigma }{\sqrt{h^{2}+a_{1}^{2}}}}$$

The 2D touch position can be specified using the ratio of the output voltage generated at each point electrode using the following equation

(6) $$\left\{\begin{array}{c} R1=\frac{V_{\mathrm{OC}}\left(4\right)}{V_{\mathrm{OC}}\left(1\right)}\approx \frac{k\frac{S\sigma }{\sqrt{h^{2}+a_{4}^{2}}}}{k\frac{S\sigma }{\sqrt{h^{2}+a_{1}^{2}}}}=\frac{\sqrt{x^{2}+y^{2}+h^{2}}}{\sqrt{{\left(l-x\right)}^{2}+{\left(l-y\right)}^{2}+h^{2}}}\\ R2=\frac{V_{\mathrm{OC}}\left(3\right)}{V_{\mathrm{OC}}\left(2\right)}\approx \frac{k\frac{S\sigma }{\sqrt{h^{2}+a_{3}^{2}}}}{k\frac{S\sigma }{\sqrt{h^{2}+a_{2}^{2}}}}=\frac{\sqrt{{\left(l-x\right)}^{2}+y^{2}+h^{2}}}{\sqrt{x^{2}+{\left(l-y\right)}^{2}+h^{2}}} \end{array}\right.$$

The ratios *R1* and *R2* obtained from the measured output voltage are plotted in Figure 5f. By solving Equation (6) using the measured voltage ratios *R1* and *R2*, we can specify the contact locations *x* and *y*. The measured spatial resolution of the tactile sensor was 0.25 cm, and the calculated sensitivity (*S =* *d* *R*/*d* *l*) was 0.21 cm^−1^, where *d* *R* is a relative change in voltage ratio and *d* *l* is a relative change in distance (Figure S18, Supporting Information). The detailed working logic flow chart for determining the touch position *x* and *y* is shown in Figure S19 of the Supporting Information.

Figure 5g depicts the output voltage as a function of the applied pressure and distance. The generated output voltage linearly increases with the increase in applied pressure because the active contact area increases at high pressures. The sensitivity (*S*) is typically defined as *S* = (*d* *V*/*V_s_*)/d*P*, where *d* *V* is the relative change in the output voltage, *V*_s_ is the saturated voltage, and *d* *P* is the relative change in touch pressure (1–600 kPa). The calculated sensitivity is 0.016 kPa^−1^, which is comparable to that of previously reported TENG‐based pressure sensors;^[^ ^24^, ^47^ ^]^ the highest‐pressure detection limit is ≈600 kPa, and the lowest pressure detection limit is ≈1 kPa. After specifying the contact position, the applied pressure can be determined by comparing the generated output voltage with respect to the applied pressure.^[^ ^31^ ^]^

The touch point and pressure were tested to ensure the proper working of the device, as shown in Figure 5h,i. The ratios for touch point 3 (2, 4) obtained from the measured output voltage were *R1* = 1.252 and *R2* = 1.252 (Figure 5h). By using the ratio graph in Figure 5f and Equation (6), two curves corresponding to the yellow curve, *R1*, and the green curve, *R2*, were plotted, and the intersection point was calculated to be (2, 3.75) using MATLAB. We can see that the experimental results and the calculated results are well matched. After the *x* and *y* positions of the touch point were determined using *R1* and *R2*, we calculated the applied pressure because the output voltage linearly increases with the touch pressure (Figure S20, Supporting Information). The distance between the touch point 3 and electrode 3 is 2.015 cm, and the output voltage is 3.26 V (Figures S20b and S21a, Supporting Information); therefore, the touch pressure is calculated to be 105 kPa. In Figure 5i, touch point 7 (1, 3) obtained voltage ratios *R1* and *R2* of 1 and 1.521, respectively. The intersection point (1.21, 2.83) was calculated using MATLAB. The distance between the touch point 7 and electrode 3 is 1.683 cm, and the output voltage is 6.83 V (Figures S20c and S21b, Supporting Information). Therefore, the touch pressure is determined to be 219 kPa using the voltage–distance–pressure relationship.

## 3 Conclusion

Herein, we report an S‐TENG in which a PVC‐gel single layer serves as an electrode as well as a dielectric material for harvesting mechanical energy and tactile sensing. The PVC‐gel‐based device possesses the following advantages.

1) High stretchability (up to the strain of 250%) and transparency (up to 91% average transmittance to the full spectrum of visible light) were achieved. By developing an S‐TENG consisting of a single layer PVC‐gel, by removing additional substrates and electrodes, the fabrication process can be simplified, and the mechanical robustness and transmittance can be improved.
2) The output performance of the S‐TENG treated with PVC‐gel is considerably improved (20 times that of native PVC) owing to the increase in the dielectric constant (190 times) and the stronger negative triboelectric properties (more negative than PFA) because of the increase in the effective work function, supported by KPFM measurements.
3) The S‐TENG based tactile sensor, demonstrated in this study, specifies the position and pressure without applying complicated electrode grid patterns using 4 small electrodes and electric displacement fields and is a passive type of sensor with low power consumption.
4) Both the PVC and DBA plasticizer used in the material are inexpensive, light, and bio‐friendly, and energy devices and tactile sensors based on them can adhere to surfaces such as the human skin. The S‐TENG with the PVC‐gel reported here provides opportunities for energy generation and tactile sensing ability for many potential applications, such as soft robots, electronic skin, organic user interfaces, and virtual reality.

In summary, we demonstrated a highly transparent, stretchable, and attachable S‐TENG, which can be used as a mechanical energy generator and tactile sensor consisting of only a single layer of plasticized PVC‐gel. Specifically, our approach of introducing plasticized PVC‐gel allows the generation of high output power, and dozens of LEDs can be operated by simple touching. In addition, the development of transparent, stretchable, and economical tactile sensors by simplifying the structure and process is expected to meet the future design requirements of tactile sensors. Finally, a smart sensor based on PVC‐gel tactile sensor will be developed by integrating a real‐time monitoring system.

## 4 Experimental Section

### Fabrication of PVC‐gel

Plasticized PVC was made from commercial PVC powder (Scientific Polymer Products, Inc., Mw 275 000, CAS: 9003‐22‐9), with THF (DAEJUNG, CAS: 109‐99‐9) as the solvent and DBA (TCI, CAS: 105‐99‐7) as a plasticizer. PVC powder was dissolved in the THF, and DBA was added to the solution and stirred for 4 h at 800 rpm. The solution was poured into a glass dish to allow the evaporation of THF at room 23 °C for 3 days, to produce a soft, gel‐like dielectric elastomer with high transparency. The different ratios of PVC:DBA was 1:0, 1:1, 1:2, 1:3, 1:5 (w/w), and the PVC‐gel thickness was 0.5 mm.

### Fabrication of the S‐TENG and the Tactile Sensor

The S‐TENG was fabricated using the as‐prepared PVC‐gel. The PVC‐gel was cut out 2.5 cm × 3 cm, and it was connected to the electric (Cu) wire for electrical connection. The tactile sensor was fabricated using the as‐prepared PVC‐gel, which was cut out 5 cm × 5 cm, and the 4‐point Ag electrodes (5 mm diameter and 50 nm thickness) was thermally evaporated under the 4 edges of the PVC‐gel. The 4 Cu wires were connected with 4 spots of the deposited Ag.

### Characterization and Measurement

The mechanical properties of the as‐prepared PVC‐gel were characterized to examine stress, strain, and Young's modulus using a universal testing machine (Tinius Olsen, H5KT) according to ASTM D638 (type V) with a crosshead speed of 50 mm min^−1^ at room temperature. Uniaxial tensile tests of the PVC‐gels were performed using dumbbell‐shaped specimens, which were cut out from the flat drop‐cast films with a thickness of 0.5 mm. The transmittance of the PVC‐gels on the ITO glass was measured using a UV–vis spectrophotometer (Optizen, 2120UV). The chemical structures of the specimens were studied using an FT‐IR (PerkinElmer, spectrometer 100). The dielectric constant was measured using a 1260 impedance per gain‐phase analyzer with a 1296 dielectric interface (Solartron Analytical Co., Farnborough, UK). The dielectric properties of the PVC‐gels were measured over a frequency range of 1 Hz to 1 MHz with a signal amplitude of 2 V at room temperature. The leakage current was measured using a potentiostat/galvanostat (Biologic Science Instruments, SP300) under an applied electric field of 80 V mm^−1^ at room temperature. For surface potential measurement, KPFM was carried out (Park Systems, XE‐100) with a Pt/Cr‐coated silicon tip and lock‐in amplifier. The output performances of S‐TENG were measured in the vertical contact mode using a 2 cm × 2 cm. The tactile sensor had an active area of 1 cm × 1 cm (contact material: nylon). The open‐circuit voltage (*V*_OC_), the short‐circuit current (*I*_SC_), and generated charge were measured using a system electrometer (KEITHLEY 6514) and an oscilloscope (Tektronix TBS2000b). Cyclic contact–separation process was applied using shaker (Labworks Inc. ET‐139), a function generator (KEYSIGHT 33210A), and linear power amplifier (Labworks Inc. PA‐138). All volunteers gave written informed consent before participating in the experiments.

## Conflict of Interest

The authors declare no conflict of interest.

## Supporting information

Supporting Information

## Data Availability

The data that support the findings of this study are available from the corresponding author upon reasonable request.
