# Supplementary material for: Plasticized PVC‐Gel Single Layer‐Based Stretchable Triboelectric Nanogenerator for Harvesting Mechanical Energy and Tactile Sensing
Source: Adv Sci (Weinh). 2022 May 26;9(22):2201070. doi: 10.1002/advs.202201070 (PMC9353411; doi:10.1002/advs.202201070)
Supplement: Supplementary file 1 — Supporting Information [file ADVS-9-2201070-s001.pdf]

ADVANCED SCIENCE Open Access

Supporting Information

for *Adv. Sci.*, DOI 10.1002/advs.202201070

Plasticized PVC-Gel Single Layer-Based Stretchable Triboelectric Nanogenerator for Harvesting Mechanical Energy and Tactile Sensing

Hyosik Park, Seung-Ju Oh, Daeyeong Kim, Mingyu Kim, Cheoljae Lee, Hyeonseo Joo, Insun Woo, Jin Woo Bae and Ju-Hyuck Lee**

Supporting Information

Plasticized PVC-gel single layer based stretchable triboelectric nanogenerator for harvesting mechanical energy and tactile sensing

Hyosik Park, Seung-Ju Oh, Daeyeong Kim, Mingyu Kim, Cheoljae Lee, Hyeonseo Joo, Insun Woo, Jin Woo Bae, Ju-Hyuck Lee**

Note S1. FT-IR characterization.

Figure S1 shows the Fourier transform infrared spectroscopy (FT-IR) spectra of only PVC, DBA, and PVC-gels. The C-Cl stretching band of PVC appeared at 635 and 692 cm^{-1} and the aliphatic ketone stretching (C=O) band of the DBA appeared at 1732 cm^{-1}. All PVC-gels showed both C-Cl stretching of PVC and the C=O band of DBA, but no new peak or shift was observed. The corresponding results indicate that a large DBA plasticizer was physically distributed in the PVC chain without forming specific intermolecular bonds.

Note S2. S-TENG working mechanism.

The operation of the S-TENG can be interpreted by the combined effect of contact electrification and electrostatic forces, which is schematically demonstrated in Figure 2b. When an external force is applied, the dielectric and PVC-gel contact each other. Consequently, the dielectric becomes positively charged, and the PVC-gel becomes negatively charged owing to its triboelectric property. The dielectric moves away from the PVC-gel surface when an external force is withdrawn. The negative charges on the PVC-gel surface repel electrons in the electrode to flow to the ground through external circuits. When the dielectric contacts the PVC-gel again, the positive charges on the dielectric balance the negative charges on the PVC-gel surface, thus, aiding the flow of electrons in the reverse direction.

Note S3. Output performance of PVC-gel based double-electrode type TENG.

Figure S4a shows a schematic of the PVC-gel based double-electrode type TENG. The TENG consisted of the PVC or PVC-gel on the indium tin oxide (ITO) coated polyethylene naphthalate (PEN) substrate for the triboelectric layer. The nylon film was on the Al electrode for the opposite triboelectric layer. The TENG had an active area of 2 cm × 2 cm, with the spacer between the PVC and PVC-gel and the top nylon film made of four springs in each corner. We measured the output performance of the TENG is a function of the DBA/PVC weight ratio ranging from 0 to 5 using a nylon film at 5 Hz and 50 kPa (Figure S4b). The result shows that the triboelectric output performance increases until the weight ratio reaches 3, reaching a maximum value of approximately 89.5 V and 8.13 μA, and then decreases with a further increase in the DBA content. The generated output voltage and current are 6.7 times higher than that of a native PVC film with an output voltage of 13.4 V and an output current of 0.7 μA and are 3.3 times higher than that of a PFA film with an output voltage of 26.9 V and an output current of 3.6 μA. The instantaneous power densities were also obtained by measuring the output current of the PVC and PVC-gel (PVC1 DBA3) TENG with an external load resistance ranging from 100 Ω to 1 GΩ, as shown in Figure S4c. The maximum output power densities of 7.3 $\mu W/cm^2$ and 137 $\mu W/cm^2$ from PVC film and PVC-gel TENG were obtained, 18-fold enhancement in output power density at 50 MΩ.

Note S4. The output performance of the S-TENG in applied pressures and frequency.

We measured the output performance of the S-TENG (PVC1 DBA3) with various applied pressures, as shown in Figure S8. The triboelectric output performance increases with applied pressure (2.5-100 kPa) because the effective contact area increases at high pressures. We also measured the output performance of the S-TENG with various frequencies, as shown in Figure S9. As the frequency increases, the output voltage does not change much. The output current increases because it is inversely proportional to the separation time, as the characteristics of a conventional TENG.

Note S5. Mechanical property of PVC-gel.

As shown in the stress-strain curves of PVC-gel with PVC:DBA ratio of 1:0 to 1:5, Young's modulus decreased (became softer) as the DBA increased (Figure 4a). The Young's modulus of PVC-gel obtained for PVC:DBA ratios of 1:0, 1:1, 1:2, 1:3, and 1:5 was 1.3 GPa, 1.6 MPa, 0.66 MPa, 0.28 MPa, and 0.14 MPa, respectively (Figure S3a). It is attributed to the plasticization of the rigid PVC chains, inducing an increase in the distance between the PVC chains and thus, decreasing the interaction force, which further enhances PVC chain mobility. It also affects the elongation at break, related to the stretchability of PVC-gels. The elongation at the break of the plasticized PVC-gels increased tremendously. However, the elongation at break (360 % for 1:1 PVC-gel, 350 % for 1:2 PVC-gel, and 250 % for 1:3 PVC-gel) decreased slightly with increasing the DBA concentration (Figure S3b). The PVC-gels consisted of disordered micro-crystallites of physically cross-linked junction points surrounded in the amorphous region. However, the addition of a large amount of DBA plasticizer to the PVC-gel can further induce thinner micro-crystallites and lower density of the physically cross-linked micro-crystallites in the PVC-gel. Consequently, such good stretchability decreased with increasing DBA contents in the PVC-gels.

Note S6. The position sensing mechanism of the S-TENG based tactile sensor.

Figure S17 shows the tactile sensor's front view and top view schematics. The open-circuit voltage based on the distances of the S-TENG can be expressed as

$$V_{OC}(n) \propto k\frac{S\sigma}{d_{En}}, \qquad n = 1,2,3,4$$

d_{En} is the distance of the contact material to the electrode 'n', and it can be expressed as

$$d_{En} = \sqrt{h^2 + a_n^2}$$

where h is the gap distance, a_n is the distance of the contact point on the PVC-gel to the electrode 'n', and it can be expressed as x and y on a 2D coordinate plane.

$$a_1 = \sqrt{x^2 + y^2}, \qquad a_2 = \sqrt{(l-x)^2 + y^2}$$
$$a_3 = \sqrt{x^2 + (l-y)^2}, \qquad a_4 = \sqrt{(l-x)^2 + (l-y)^2}$$

l is the length of the tactile sensor. Thus, the open-circuit voltage of the E1 to E4 can be expressed as

$$V_{OC}(1) \propto k\frac{S\sigma}{d_{E1}} = k\frac{S\sigma}{\sqrt{h^2 + x^2 + y^2}}, \qquad V_{OC}(2) \propto k\frac{S\sigma}{d_{E2}} = k\frac{S\sigma}{\sqrt{h^2 + (l-x)^2 + y^2}}$$
$$V_{OC}(3) \propto k\frac{S\sigma}{d_{E3}} = k\frac{S\sigma}{\sqrt{h^2 + x^2 + (l-y)^2}}, V_{OC}(4) \propto k\frac{S\sigma}{d_{E4}} = k\frac{S\sigma}{\sqrt{h^2 + (l-x)^2 + (l-y)^2}}$$

According to the above equation, the ratio of the open-circuit voltage of $V_{OC}(1)$ to $V_{OC}(4)$ and $V_{OC}(2)$ to $V_{OC}(3)$ can be expressed as

$$\begin{cases} R1 = \dfrac{V_{OC}(4)}{V_{OC}(1)} \approx \dfrac{k\dfrac{S\sigma}{\sqrt{h^2 + (l-x)^2 + (l-y)^2}}}{k\dfrac{S\sigma}{\sqrt{h^2 + x^2 + y^2}}} = \dfrac{\sqrt{h^2 + x^2 + y^2}}{\sqrt{h^2 + (l-x)^2 + (l-y)^2}} \\ R2 = \dfrac{V_{OC}(3)}{V_{OC}(2)} \approx \dfrac{k\dfrac{S\sigma}{\sqrt{h^2 + x^2 + (l-y)^2}}}{k\dfrac{S\sigma}{\sqrt{h^2 + (l-x)^2 + y^2}}} = \dfrac{\sqrt{h^2 + (l-x)^2 + y^2}}{\sqrt{h^2 + x^2 + (l-y)^2}} \end{cases}$$

We can specify the contact locations x and y by solving the above equation.

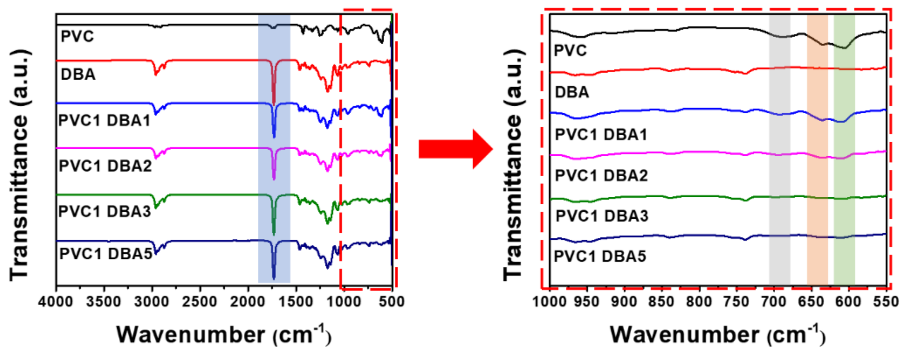

Figure S1. FT-IR spectra of the PVC, DBA, and PVC-gels and their partially enlarged FT-IR spectra at 550 cm^{-1} to 1000 cm^{-1}.

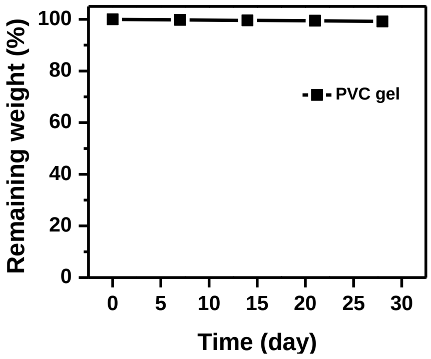

Figure S2. Remaining weight % of DBA plasticizer in PVC-gel at room temperature for 30 days.

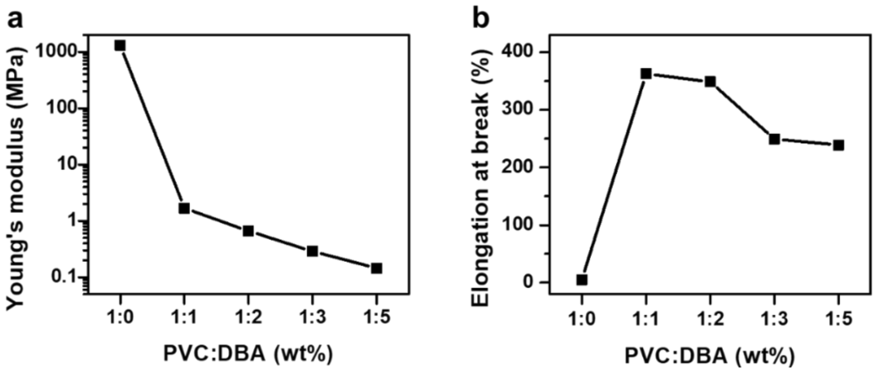

Figure S3. Mechanical properties of PVC-gel. a) Young's modulus and b) Elongation at break (%) with the change in the PVC:DBA ratio (wt %).

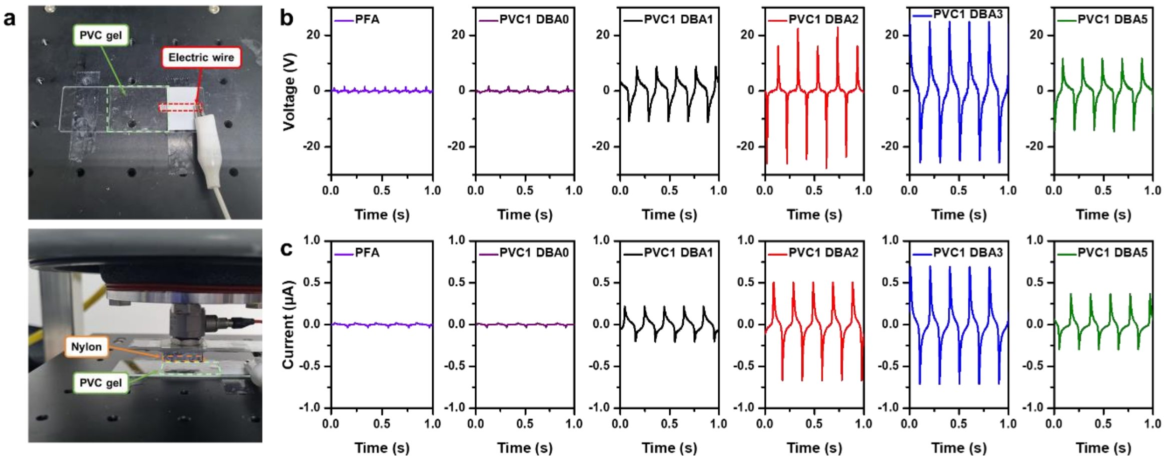

Figure S4. The output performance of S-TENG. a) Photographs of S-TENG. b) Output voltage signals and c) current signals in the S-TENG based PFA, PVC, and PVC-gels by nylon film contact.

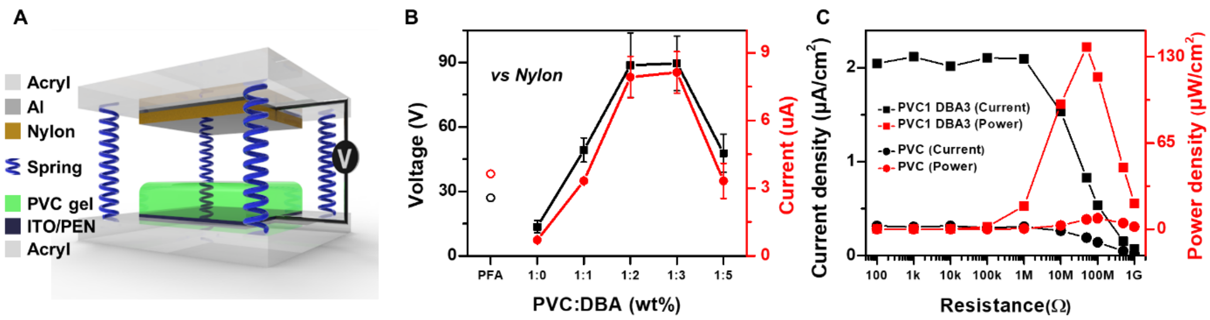

Figure S5. PVC-gel based double-electrode type TENG. a) Schematic of double-electrode type TENG based PFA, PVC, and PVC-gels by nylon film contact. b) Output voltage and current with the change in the PVC:DBA ratio (wt %). c) Output current density and power density of the PVC and PVC1 DBA3 on external loading resistance.

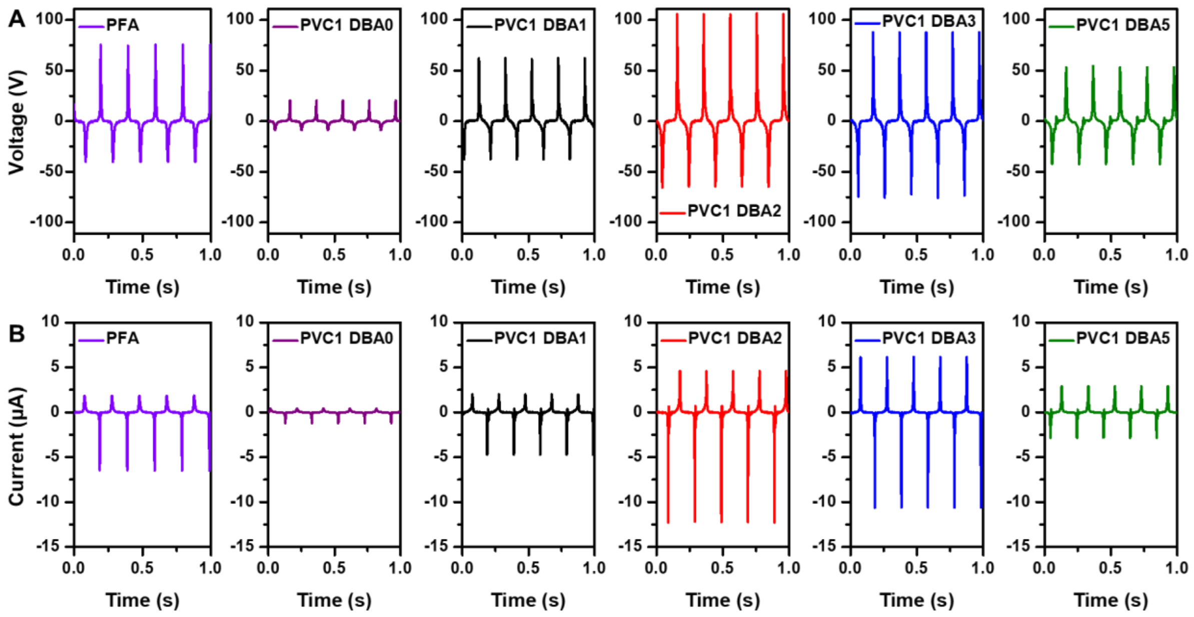

Figure S6. The output performance of double-electrode type TENG. a) Output voltage signals and b) current signals in double-electrode type TENG based PFA, PVC, and PVC-gels by nylon film contact.

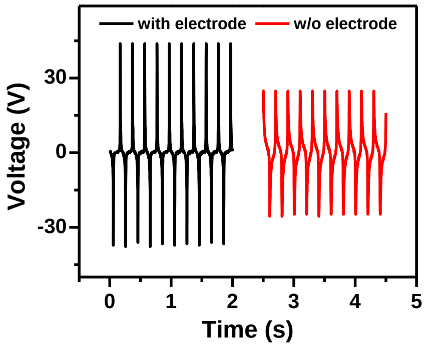

Figure S7. The output voltage of PVC-gel TENG with and without electrode.

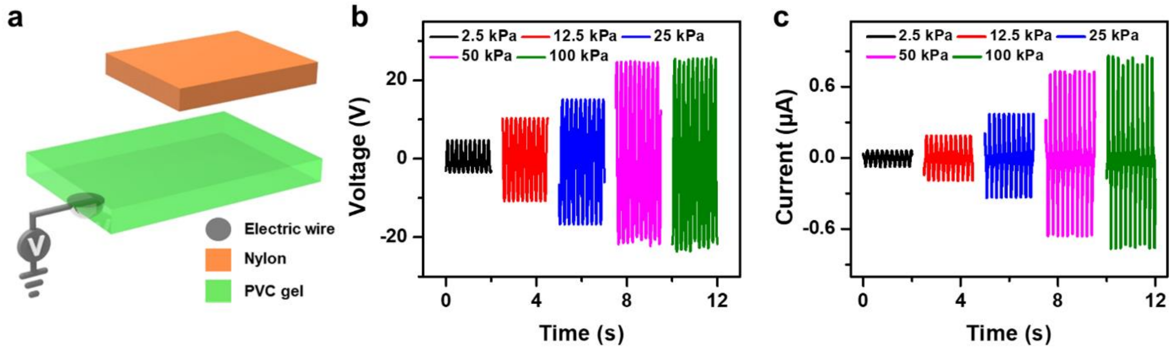

Figure S8. The output performance in S-TENG for various applied pressures (2.5 kPa to 100 kPa) at 5 Hz and active area of 2 cm × 2 cm. a) Schematic of the PVC-gel (PVC1 DBA3) based S-TENG. b) Output voltage and c) current.

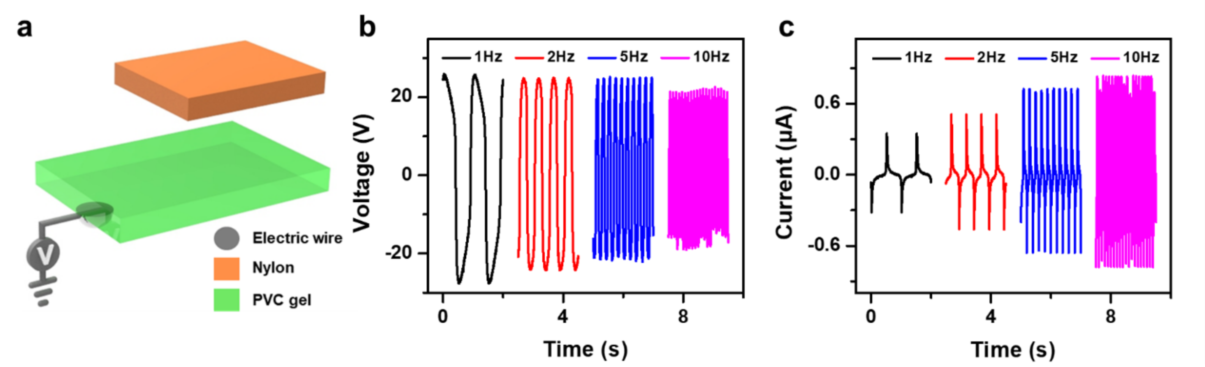

Figure S9. The output performance in S-TENG for varying frequencies (1 Hz to 20 Hz) at 50 kPa and active area of 2 cm × 2 cm. a) Schematic of the PVC-gel (PVC1 DBA3) based S-TENG. b) Output voltage and c) current.

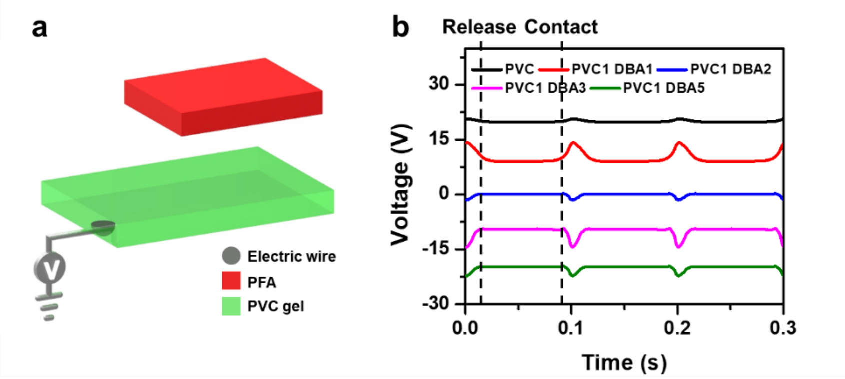

Figure S10. Triboelectricity of PVC-gels by PFA film contact. a) Schematic of S-TENG (Top part: PFA, Bottom part: PVC or PVC-gel) to compare triboelectricity. b) PVC and PVC1 DBA1 generate the positive output when they contact PFA, but PVC1 DBA2, PVC1 DBA3, and PVC1 DBA5 generate the negative output when they contact with PFA.

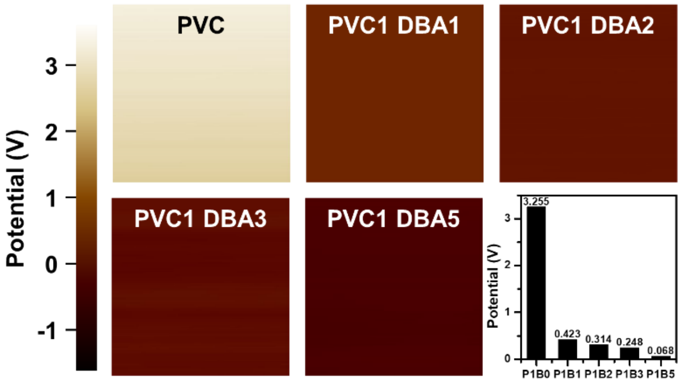

Figure S11. KPFM surface potential of PVC and PVC-gel.

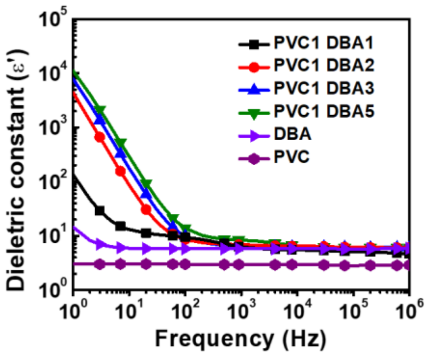

Figure S12. The dielectric constant of PVC, DBA, and PVC-gels at 1 Hz to 1 MHz.

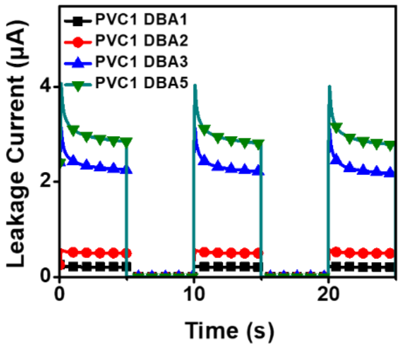

Figure S13. Leakage current of PVC-gels.

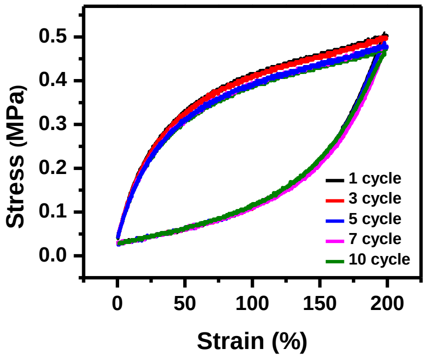

Figure S14. Stress-strain hysteresis loops of the cyclic test (10 cycles) for PVC1 DBA3.

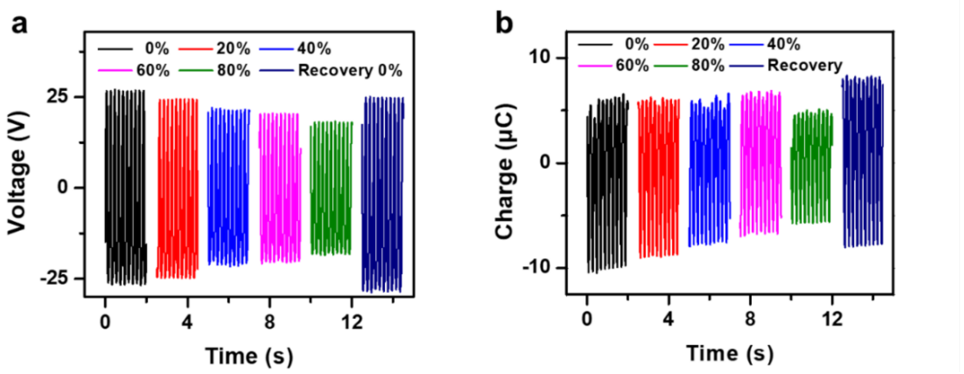

Figure S15. Output performance by varying the strain from 0 % to 80 % and recovered PVC-gel (PVC1 DBA3) based on the S-TENG. a) Output voltage and b) charge.

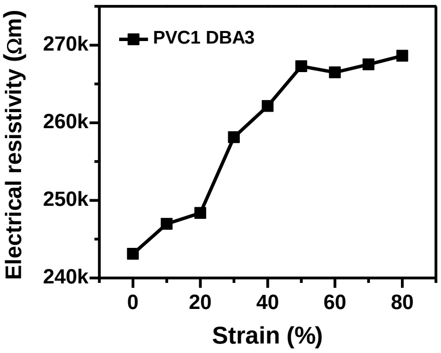

Figure S16. Electrical resistivity of PVC1 DBA3 film in a stretched state (0, 20, 40, 60, and 80 %).

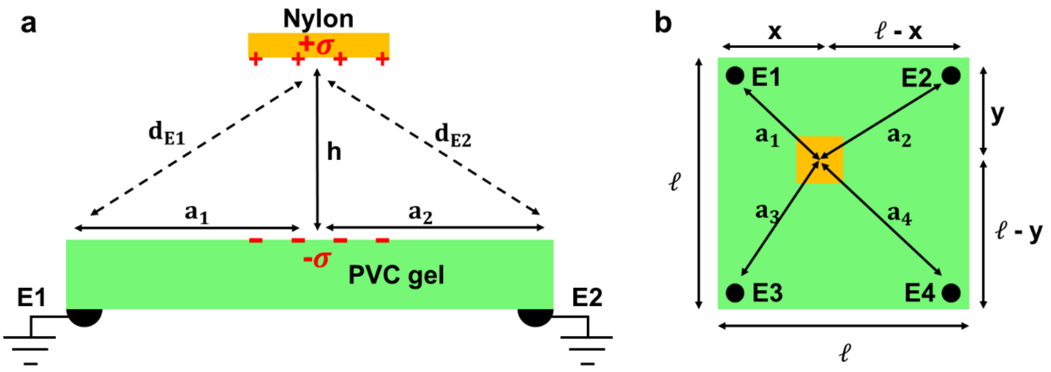

Figure S17. The schematic of the tactile sensor. a) Front view and b) top view.

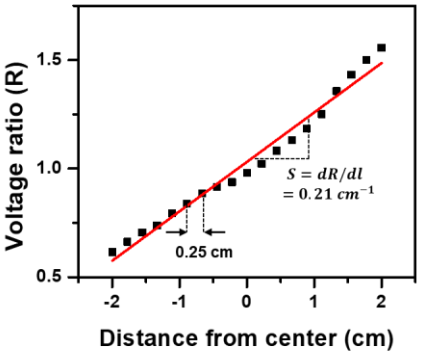

Figure S18. Spatial resolution and sensitivity of the PVC-gel tactile sensor.

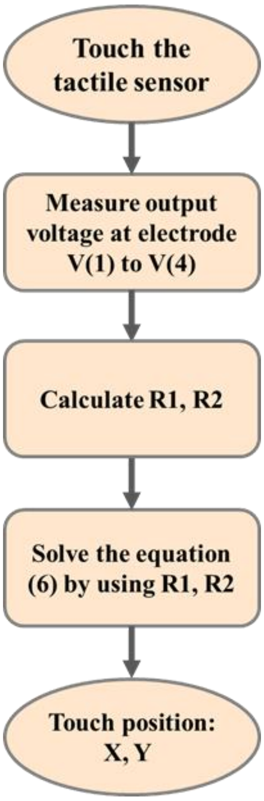

Figure S19. Working logic flow chart for determining the touch position.

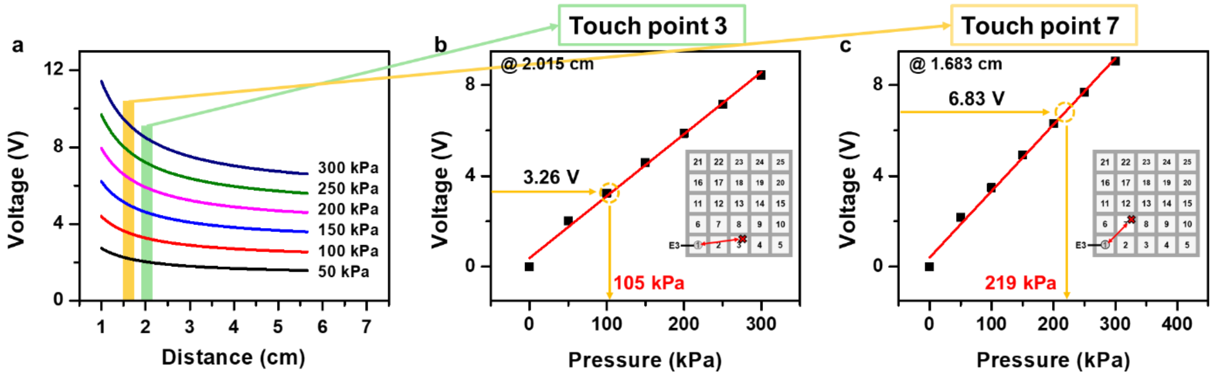

Figure S20. The pressure-sensing mechanism of the tactile sensor. a) Distance-voltage for various applied pressures. b) and c) Pressure sensing algorithm for touch point 3 (105 kPa) and touch point 7 (219 kPa).

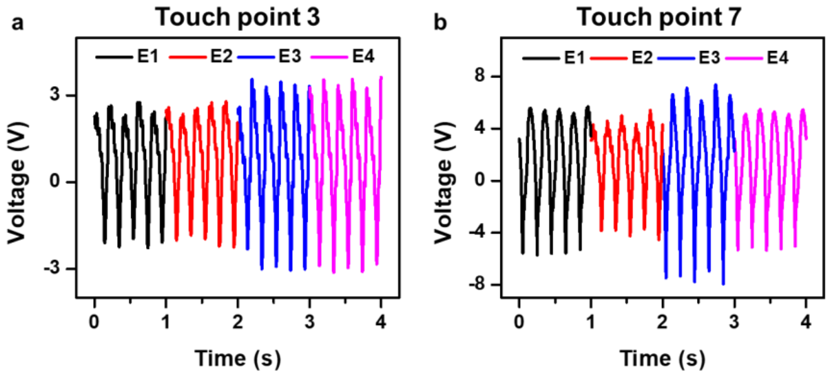

Figure S21. Output voltage signals of each electrode (E1 to E4). a) Touch point 3 and b) touch point 7.
